# Occupational Benzene Exposure in the Norwegian Offshore Petroleum Industry, 2002–2018

**DOI:** 10.1093/annweh/wxac022

**Published:** 2022-05-06

**Authors:** Hilde Ridderseth, Dagrun Slettebø Daltveit, Bjørg Eli Hollund, Jorunn Kirkeleit, Hans Kromhout, Kirsti Krüger, Liv-Torill Austgulen, Magne Bråtveit

**Affiliations:** Department of Global Public Health and Primary Care, University of Bergen, Bergen, Norway; Department of Global Public Health and Primary Care, University of Bergen, Bergen, Norway; Department of Global Public Health and Primary Care, University of Bergen, Bergen, Norway; Department of Global Public Health and Primary Care, University of Bergen, Bergen, Norway; Institute for Risk Assessment Sciences, Utrecht University, CS Utrecht, The Netherlands; Equinor ASA, Stavanger, Norway; Equinor ASA, Stavanger, Norway; Department of Global Public Health and Primary Care, University of Bergen, Bergen, Norway

**Keywords:** cancer, determinants, full-shift exposure, job group, occupational benzene exposure, offshore installation, petroleum industry, sampling duration, time trend

## Abstract

**Purpose:**

Workers on offshore petroleum installations are at risk of being exposed to benzene which is carcinogenic to humans. The present study aimed to assess the time trend of full-shift benzene exposure from 2002 to 2018 in order to characterize benzene exposure among laboratory technicians, mechanics, process operators, and industrial cleaners, and to examine the possible determinants of benzene exposure.

**Methods:**

A total of 924 measurements of benzene exposure from the Norwegian petroleum offshore industry were included. The median sampling duration was 680 min, ranging from 60 to 940 min. The overall geometric mean (GM) and 95% confidence interval, time trends, and determinants of exposure were estimated using multilevel mixed-effects tobit regression analyses. Time trends were estimated for sampling duration below and above 8 h, both overall and for job groups. The variability of exposure between installation and workers was investigated in a subset of data containing worker identification.

**Results:**

The overall GM of benzene exposure was 0.004 ppm. When adjusting for job group, design of process area, season, wind speed, and sampling duration, industrial cleaners had the highest exposure (GM = 0.012). Laboratory technicians, mechanics, and process operators had a GM exposure of 0.004, 0.003, and 0.004 ppm, respectively. Overall, the measured benzene exposure increased by 7.6% per year from 2002 to 2018. Mechanics had an annual increase of 8.6% and laboratory technicians had an annual decrease of 12.6% when including all measurements. When including only measurements above 8 h, mechanics had an increase of 16.8%. No statistically significant time trend was found for process operators. Open process area, high wind speed, and wintertime were associated with reduced exposure level.

**Conclusions:**

An overall increase in measured exposure was observed from 2002 to 2018. The increase may reflect changes in measurement strategy from mainly measuring on random days to days with expected exposure. However, the time trend varied between job groups and was different for sampling duration above or below 8 h. Industrial cleaners had the highest exposure of the four job groups while no differences in exposure were observed between laboratory technicians, mechanics, and process operators. The design of the process area, job group, wind speed, and season were all significant determinants of benzene exposure.

**What’s important about this paper**

Workers on petroleum offshore installations can be exposed to benzene which is carcinogenic. This study describes recent (2002–2018) exposure measurements, providing updated knowledge about benzene exposure levels in this industry that can support epidemiological studies and exposure assessments. Laboratory technicians, mechanics, process operators, and industrial cleaners are job groups exposed to benzene on petroleum offshore installations.

## Introduction

Exposure to benzene among workers on offshore oil and gas installations may occur during the operation and maintenance of process system. The process stream comprises crude oil, natural gas, condensate, and produced water containing varying concentrations of benzene. Process operators, laboratory technicians, mechanics, and industrial cleaners are among the highest exposed job groups on these installations (Steinsvåg *et al.*, 2007).

Benzene is classified as carcinogenic to humans (Group 1) by The International Agency for Research on Cancer (IARC, 2018) and an excess risk of leukaemia associated with exposure to benzene has been reported among workers in the petroleum industry (Glass *et al.*, 2003; Stenehjem *et al.*, 2015). The occupational exposure limit value (OELV) in most high-income countries ranges from 0.2 to 1.0 ppm as an 8-h average (ECHA, 2018; IARC, 2018). Recently, the EU Committee for Risk Assessment (RAC) proposed a new limit for benzene of 0.05 ppm as an 8-h time-weighted average. The new limit is assumed to protect the workers from induced chromosome damage and haematological suppression where peripheral blood cell types are affected. As a result, there will be no significant residual cancer risk (RAC, 2018). In Norway, the OELV was reduced from 1.0 to 0.2 ppm on 1st July 2021.

In the Norwegian offshore petroleum industry (1994–2003), the overall geometric mean (GM) exposure to benzene among process operators, laboratory technicians, deck crews, and electricians was reported to be 0.007 ppm [range: less than the limit of detection (LOD) to 2.6 ppm], sampling duration 7–8 h (Steinsvåg *et al.*, 2007). In the Canadian land-based conventional oil and gas industry from 1985 to 1996, Verma *et al.* (2000) reported a GM benzene exposure of 0.036 mg m^−3^ (range: 0.010–7.78 mg m^−3^ [0.011 (0.003–2.396) ppm]), sampling duration 7.5–12 h. Under normal operation conditions on an offshore installation in 2005, process operators had a GM exposure of 0.005 ppm (range: <0.001–0.69 ppm) (Bråtveit *et al.*, 2007), while workers maintaining equipment placed inside tanks containing residues of crude oil had a GM of 0.15 ppm (range: 0.01–0.62 ppm), sampling duration 4–16 h (Kirkeleit *et al.*, 2006).

Occupational benzene measurements from operation and maintenance work performed after 2005 in the petroleum offshore industry have not been published. To establish whether benzene exposure in this industry has changed over the last two decades, more recent measurements of benzene exposure levels are needed.

Based on reports from 2002 to 2018, comprising 924 personal measurements of benzene exposure provided by two major operators on the Norwegian Continental Shelf, we aimed to assess the time trends of benzene exposure, overall, and for laboratory technicians, mechanics, and process operators separately. For these job groups and for industrial cleaners, we estimated GM exposure and identified the potential determinants of benzene exposure.

## Methods

### Dataset

**Figure 1.**
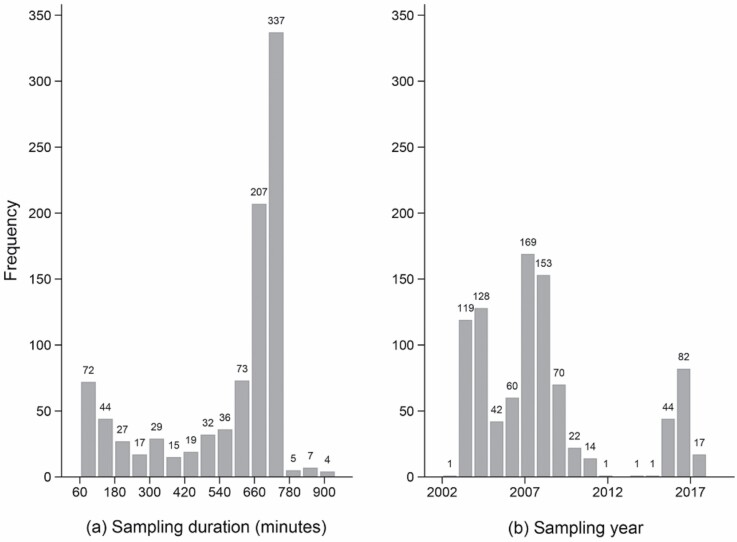
Frequency of the available personal air measurements of benzene categorized by (a) sampling duration and (b) sampling year.

This project included 98 reports containing a total of 924 personal measurements of benzene exposure among workers collected on 25 offshore oil and gas installations on the Norwegian Continental Shelf. The number of measurements per installation varied between 4 and 118. The reports were mainly from two of the major oil and gas operating companies, which merged into one company in 2007. The measurements were conducted by or were under the guidance of occupational hygienists. The data were available from both hardcopy reports and a database previously developed by the University of Bergen (UiB) by Steinsvåg *et al.* (2007) and Bråtveit *et al.* (2012). This database contained 350 personal measurements collected between 2002 and 2006 and has been supplemented by 574 new measurements collected between 2007 and 2018. Relevant information from the reports was extracted and transferred to the database. Personal air measurements with a sampling duration of 60–940 min were included in this study (Fig. 1a). Stationary measurements and measurements conducted inside a breathing mask were excluded. The benzene concentration was recorded as parts per million (ppm). When the measurement results in the reports were given as milligram per cubic metre (mg m^−3^), the following conversion formula was used: (mg m^−3^ × 24.06)/78.11 mol.

In order to understand changes in benzene exposure during the sampling period (2002–2018), we grouped the measurements into three sampling periods due to different chemical exposure projects focused on benzene exposure in the industry. The first period (2002–2006, *n* = 350) mainly comprised measurements presented in papers from Kirkeleit *et al.* (2006), Bråtveit *et al.* (2007), and Steinsvåg *et al.* (2007). The second period (2007–2011, *n* = 428) comprised measurements performed during the project ‘Chemical working environment in the oil and gas industry’ implemented by the Norwegian Oil and Gas Association (2011). The third period (2012–2018, *n* = 146) contained benzene measurements from compliance measurements initiated by the installations (Fig. 1b).

A worker identification code was reported in 31% (289/924) of the benzene measurements, which were mainly performed from 2003 to 2008. Out of 289 measurements that had worker identification, 250 (87%) were repeated measurements (2–10 measurements per subject) from 73 unique workers. Most of these measurements were collected on 2–3 consecutive days, while a few were repeated over months.

### Potential determinants of exposure

Contextual information on potential determinants were extracted from the reports and their relevance was assessed by the authors. The following potential determinants of full-shift benzene exposure were considered as relevant: job group, design of process area, season, wind speed, temperature, and year of production start (installation age).

Four distinct job groups comprising laboratory technicians, mechanics, process operators, and industrial cleaners were defined in the study based on the grouping used in previous study by Steinsvåg *et al.* (2007). The number of measurements was also crucial for the selection of job groups. Due to the limited number of benzene measurements in the remaining job groups, instrument technicians, deck crews, derrickman, and tank inspectors were merged into one group named ‘Others’. The group of ‘Others’ has been included in the analysis but will not be discussed further. The four job groups performed different tasks with the potential for benzene exposure during their 12-h shifts. Laboratory technicians collect samples on a daily basis from the process stream outdoors and analyse the samples in the laboratory. Process operators, the largest job group in the operator companies, control the process and the equipment in the process area on a regular basis. They collect samples from the process stream outdoors but less frequently than laboratory technicians. Other work tasks with the potential for benzene exposure includes, as for example, changing or cleaning different types of filters. In addition, the process operators control gas freeing, before other workers, such as mechanics, and split and disassemble equipment. Mechanics disassemble equipment prior to maintenance or repair and subsequently re-assemble it. The level of exposure might depend on how they prepare to open the process system. Flushing with water and ventilating of opened equipment before a maintenance task could reduce exposure. Mechanics mainly work outdoors in the process and utility areas, although they occasionally bring equipment indoors to mechanical workshops for further work (Bråtveit *et al.*, 2012; Ridderseth *et al.*, 2019). Industrial cleaners work in multiple types of storage and process tanks. They prepare to open the tanks before cleaning. In addition, they serve as rescue service personnel for other cleaners working inside the tank.

The seasons were divided into summer (April to September) and winter (October to March) in order to examine seasonal differences in exposure (Steinsvåg *et al.*, 2005). Wind may affect the level of exposure when work is performed outdoors. Wind speed was reported in 35% of the measurements. If wind speed at the oil fields was not reported, it was collected from the Norwegian Meteorological Institute (https://www.yr.no) and recorded as median wind speed for the day that the measurements were performed. The wind speed was divided into eight groups using the Beaufort Scale, then into three groups: light air 0–3.9 m s^–1^, breeze 4.0–11.9 m s^−1^, and gale 12.0–20.7 m s^–1^. In addition, the median air temperature from the measurement day was collected from the Norwegian Meteorological Institute and presented as a continuous variable

Exposure to benzene is mainly related to the process area but can also occur in drilling area where 18 of the measurements were taken. The installations have major differences in the design of the process area, which may affect exposure to benzene. Consequently, they were divided into three groups: open areas if the process areas had no walls, partially restricted areas if the process areas had partial walls, and restricted areas, if the process areas were located within walls (Det Norske Veritas Germanischer Lloyd (DNVGL), 2016). The installations were built between 1979 and 2005 and the design of the platform and equipment has changed over the years. In early years, the installations were built more closed than in later years. We divided the installation into three groups based on the year of construction: 1979–1989, 1990–1999, and 2000–2005.

### Other variables

**Figure 2.**
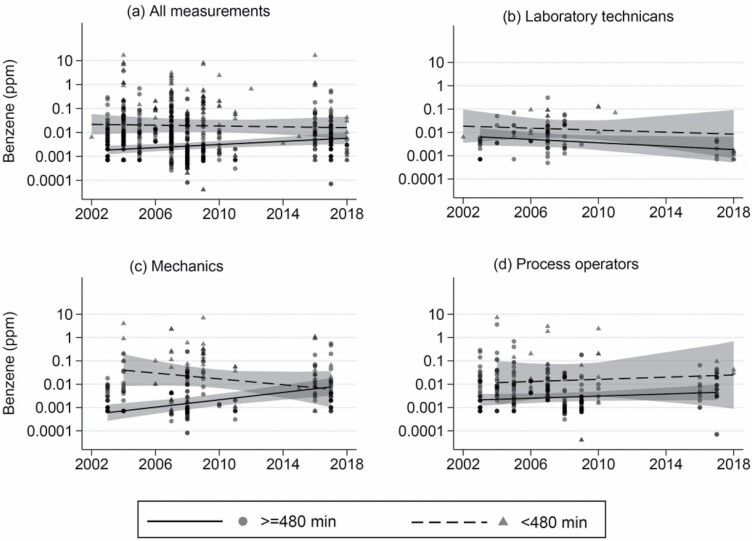
Unadjusted time trend for (a) all measurements, (b) laboratory technicians, (c) mechanics, and (d) process operators. Annual GM including CI for benzene exposure in parts per million (ppm), from 2002 to 2018. Sampling duration above 479 min and below 480 min. The spline goes through two knots (2007 and 2012). The colour intensity of the dots indicates the number of measurements; the darker colour has a higher number of measurements than the lighter colour.

To investigate the effect of the sampling duration on the measured exposure level, we split the measurements into two groups: below and above an 8-h sampling duration (Fig. 2). An offshore work shift normally lasts for 12 h, including a 1.5-h break, meetings, and administrative work. Thus, we assume that measurements lasting 8 h or more are representative of full-shift exposure.

Sampling methods for benzene have changed over the years, and different methods may have influenced the reported benzene concentration. Four methods were represented in the dataset: 3M dosimeter (passive), automated thermal desorption (ATD) (passive), charcoal tubes (active), and ATD (active). When the sampling duration is short, an active sampling method is usually selected to avoid measurements below the LOD. A passive sampling method is normally used for a longer sampling duration. Due to the correlation between sampling method and sampling duration, the sampling method was excluded as an adjustment variable in the exposure models (Table 3) but included as a variable in the unadjusted analyses (Table 1).

**Table 1.** Descriptive data of the variables job group, design of process area, wind speed, season, and sampling method by using mixed-effects models.

Variables	*N*	*N* (%) <LOD	AM (95% CI) (ppm)	Max (ppm)	GM (95% CI) (ppm)
Overall	924	244 (26)	0.075 (0.041–0.138)	16.75	0.004 (0.003–0.006)
Job group					
Laboratory technicians	102	28 (27)	0.057 (0.029–0.112)	0.31	0.004 (0.002–0.007) (ref.)
Mechanics	279	74 (27)	0.050 (0.029–0.091)	7.00	0.004 (0.002–0.006)
Process operators	353	110 (31)	0.049 (0.028–0.087)	7.28	0.003 (0.002–0.005)
Industrial cleaners	55	11 (20)	0.315 (0.132–0.753)	1.51	0.022 (0.010–0.048)*
Other	135	21 (16)	0.142 (0.069–0.257)	16.75	0.009 (0.005–0.016)
Design of process area					
Open	124	38 (31)	0.025 (0.010–0.060)	3.04	0.002 (0.001–0.004) (ref.)
Partially restricted	426	78 (18)	0.087 (0.049–0.192)	16.75	0.006 (0.003–0.011)*
Restricted	374	128 (34)	0.081 (0.039–0.167)	16.50	0.005 (0.003–0.010)*
Wind speed					
Light air (0–3.9 m s^−1^)	91	4 (6)	0.078 (0.038–0.162)	2.4	0.004 (0.002–0.008)
Breeze (4.0–11.9 m s^−1^)	634	154 (25)	0.087 (0.049–0.156)	16.75	0.005 (0.004–0.008) (ref.)
Gale (12.0–20.0 m s^−1^)	199	86 (43)	0.033 (0.017–0.062)	3.70	0.002 (0.001–0.003)*
Season					
Winter	523	166 (32)	0.060 (0.032–0.112)	16.75	0.003 (0.002–0.006) (ref.)
Summer	401	78 (19)	0.088 (0.047–0.162)	8.00	0.005 (0.003–0.008)*
Sampling method					
3M dosimeter	682	213 (31)	0.043 (0.023–0.080)	16.75	0.003 (0.002–0.004) (ref.)
ATD active	24	5 (21)	0.409 (0.139–1.200)	21.4	0.026 (0.009–0.071)*
ATD passive	148	14 (9)	0.098 (0.047–0.203)	4.0	0.006 (0.003–0.011)*
Charcoal active	70	12 (17)	0.238 (0.108–0.524)	3.04	0.015 (0.008–0.030)*

*N*, number of measurements; ref., reference group.

*Statistically significant different (*P* < 0.05) compared to the reference group.

**Table 3.** Random effect model (Model 0) of log-transformed data (ppm) and mixed-effects models including the fixed effects: job group, design of process area, season, wind speed, and sampling duration. Model 2 includes sampling year in addition to the other fixed effects.

	Model 0	Model 1	*P*-value
	β (SE)	β (SE)	
Intercept	−5.49 (0.22)	−3.71 (0.44)	
Job group			
Laboratory technicians		ref.	
Mechanics		−0.41 (0.26)	0.116
Process operators		0.01 (0.26)	0.971
Industrial cleaners		0.84 (0.38)	0.029
Other		0.09 (0.30)	0.761
Design of process area			
Open		ref.	
Partially restricted		0.98 (0.37)	0.008
Restricted		0.72 (0.38)	0.060
Season			
Winter		ref.	
Summer		0.31 (0.16)	0.056
Wind speed			
Light air (0–3.9 m s^−1^)		0.15 (0.24)	0.519
Breeze (4.0–11.9 m s^−1^)		ref.	
Gale (12.0–20.0 m s^−1^)		−0.74 (0.19)	0.008
Sampling duration			
Minutes (continuous)		−0.004 (0.0003)	0.000
Between-installation variance (_bp_S^2^)	0.90 (0.34)	0.27 (0.13)	
Within-installation variance (_wp_S^2^)	4.89 (0.27)	3.93 (0.22)	
Total variance (_bp_S^2^ + _wp_S^2^)*a*	5.79	4.2	
% Variance explained by fixed effects^*b*^		28	

β, intercept. Total variance _(random effects)_ – Total variance _(fixed effects)_ * 100/Total variance _(random effects)._

^*ª*^Total variance = bpS^2^ + bwS2.

^*b*^% reduction in variance from random effect model to the respective mixed-effects models.

### Limit of detection

The measurements in the database contained left-censored data below the LOD. The LOD was specified in most reports but was missing for 11 measurements. These measurements were replaced by a fixed LOD, generated by using the LOD from a similar analysis from the same year, laboratory, and sampling duration. The LOD ranged from 0.001 to 0.09 ppm depending on sampling duration, sampling method (active or passive), and laboratory.

### Statistical analysis

Due to the right-skewed distribution of the dataset, the measured values were log-transformed. Multilevel mixed-effects tobit regression was chosen in order to take multiple left-censored measurements into account. To account for repeated measurements taken from the same installation, the individual installation was viewed as a random effect. In our data, 26% of the measurements were below the LOD. The tobit regression model assumes that the distribution of the censored data follows the distribution of the data above the LOD (Helsel, 2005; Lotz *et al.*, 2013).

Multilevel mixed-effects tobit regression was used to estimate descriptive data as GM and GM confidence interval (CI). Based on the method described by Almerud *et al.* (2017), we estimated the arithmetic mean (AM) and 95% CI. The variables that included subgroups with a *P*-value <0.05 are given in Table 1. The effect of temperature and the year of production start were not statistically significant and were therefore excluded from Table 1 and further analyses.

To study the time trends overall, all five job groups were included. Then, we study time trend for each job group. We performed a multilevel mixed-effects tobit regression analyses of the outcome in which the sampling year was included as a continuous fixed effect. Thirteen models were performed, including unadjusted and adjusted models (adjusted for design of process area, season, wind speed, and sampling duration), i.e. for sampling duration below and above 8 h, both overall and for each job group. The time trend for industrial cleaners was not estimated due to measurements only being available for 2004 and 2007. The unadjusted time trend was visualized in a scatter plot and linear splines including a 95% CI for sampling duration both below and above 8 h. In the graph, measurements below the LOD were imputed as $LOD/\sqrt{2\;\;\;}$. Linear splines were plotted with two knots, 2007 and 2012 (Fig. 2), due to the three sampling periods as previously described.

To identify determinants of exposure, we developed first a random effects model with between- and within-installation variability. The next was multilevel mixed-effects tobit regression model including job group, season, design of process area, wind speed, and sampling duration as fixed effects. Only variables with *P*-value <0.10 in one of the subgroups were included in the model. The total variance was estimated by adding the variance between installations (_bp_S^2^) and within installations (_ww_S^2^). The percentage reductions in the exposure variability between and within installation due to including fixed factors were estimated.

A subset of the data containing measurements with worker identification numbers (*n* = 294) was used to investigate the variability of exposure within and between workers. First, we performed an unadjusted nested two-level random effects model, where we included worker’s identification number and offshore installation as random effects. To evaluate season, wind speed, design of process area, and job group’s influences on the variance estimates (within-worker, between-worker within-installation, and between-installation), we included these variables separately in four models. We then combined the four variables as fixed effects in a fifth model. The total variance was estimated by adding the variance between installations (_bp_S^2^), between worker within installation (_bw_S^2^), and within worker (_ww_S^2^). The percentage reduction in variance estimates from the naive models (with just random effects) was estimated for the fixed factors: season, wind speed, design of process area, job group separately, and for all four combined. The percentage was calculated as follows:


$$bpS^{2}\left(\text{random effects model}\right)-bpS^{2}\left(\text{mixed effects model}\right)/bpS^{2}\left(\text{random effects model}\right)*100,$$

where _bp_S^2^ is between-installations variance. Analogous calculations were conducted for between-worker within-installation (_bw_S^2^), within-worker (_ww_S^2^), and total variance (_bp_S^2^ + _bw_S^2^ + _ww_S^2^).

## Results

The overall GM benzene exposure on the 25 offshore installations from 2002 to 2018 was 0.004 ppm (95% CI 0.003–0.006) with an AM of 0.075 ppm (95% CI 0.041–0.138). The measurements ranged from GM below the LOD to 16.8 ppm (Table 1). The benzene exposure among industrial cleaners was 0.022 ppm, while laboratory technicians, mechanics, and process operators were exposed to 0.004, 0.004, and 0.003 ppm, respectively. Furthermore, level of exposure varied according to the design of the process area, wind speed, season, and sampling method.

### Time trend

The benzene exposure varied considerably across the sampling years (Fig. 2a). A visualization of the unadjusted exposure over the years is presented in a scatter plot including all measurements divided into below and above an 8-h sampling duration. A linear spline with two knots in 2007 and 2012 and the corresponding CI were estimated for each of the two groups of sampling duration (Fig. 2).

When adjusting for the variables job group, design of process area, season, wind speed, and sampling duration, the overall annual time trend was statistically significant at 7.6% (95% CI 2.9–12.5%) (Table 2). Measurements with a sampling duration of 8 h or more showed an annual increase in exposure of 9.2% (95% CI 4.0–14.8) while measurements with a sampling duration of less than 8 h did not have statistically significant changes in time trend (0.9%, 95% CI −9.4 to 12.3). Further, laboratory technicians’ exposure decreased statistically significant by 12.6% (95% CI −20.0 to −4.8), when all measurements were included. For mechanics, a statistically significant increase of 8.6% was observed when including all measurements, and 16.8% (95% CI 6.7–27.7) when including measurements above an 8-h sampling duration. For the process operators, there was no statistically significant time trend.

**Table 2.** Annual percentage change in GM benzene exposure overall, by sampling duration and within job groups. Unadjusted models and models adjusted for the variables design of process area (open, partially restricted, restricted), season (summer, winter), wind speed (three categories), and sampling duration (60–940 min). In addition, ‘All measurements’ were adjusted for job group (five groups).

		Unadjusted	Adjusted
	Number of measurements	Annual percent change in GM (95% CI)	Annual percent change in GM (95% CI)
All measurements	924	12.1 (6.4–18.1)	7.6 (2.9–12.5)
Measurements ≥ 480 min	696	7.9 (2.7–13.3)	9.2 (4.0–14.8)
Measurements < 480 min	228	−2.0 (−12.0 to 9.2)	0.9 (−9.4 to 12.3)
Laboratory technicians	102	−8.5 (−17.3 to 1.2)	−12.6 (−20.0 to −4.8)
Measurements ≥ 480 min	79	−8.0 (−17.8 to 2.9)	−11.2 (−18.4 to 2.11)
Measurements < 480 min	23	−4.8 (−24.4 to 19.8)	−14.0 (−32.4 to −9.1)
Mechanics	279	10.9 (1.4–21.3)	8.6 (1.0–16.7)
Measurements ≥ 480 min	198	19.9 (9.8–31.0)	16.8 (6.7–27.7)
Measurements < 480 min	80	−13.4 (−30.7 to 8.2)	3.8 (−10.1 to 19.8)
Process operators	353	10.9 (2.6–20.0)	6.0 (−1.6 to 14.2)
Measurements ≥ 480 min	323	5.4 (−2.2 to 13.6)	4.2 (−3.1 to 12.1)
Measurements < 480 min	30	5.6 (−23.1 to 45.1)	1.2 (−23.5 to 34.0)
Others	135	16.0 (−4 to 40.2)	13.0 (1.8–27.0)

### Determinants of exposure

The tobit mixed-effects model including job group, season, design of process area, wind speed, and sampling duration as fixed effects explained 28% of the total variability in benzene exposure (Table 3; Model 1). Industrial cleaners had a statistically significant higher exposure than laboratory technicians, while there was no difference in exposure between the laboratory technicians and mechanics or process operators.

The design of the process area was a significant determinant of benzene exposure. Restricted installations increased exposure by around two times (e^0.72^) compared to open installations (Table 3; Model 1). Partially restricted installations were associated with around 2.5 times higher exposure compared to open installations.

The wind speed differed from summer to winter; the median of wind speed in summer was 7.2 m s^−1^ and in winter 9.6 m s^−1^. The effect of the wind speed on the exposure was significant for gale (12–20.7 m s^−1^), the exposure was 46% less than exposure during breeze (4.0–11.9 m s^−1^). Exposure to benzene during the summer season was 1.5 times higher than that during the winter season even when adjusting for wind speed (Table 3; Model 1).

Sampling durations affected the measured exposure; a shorter sampling duration was associated with higher exposure (Table 3; Model 1). Figure 2 shows how measurements with a sampling duration below 8 h are associated with a higher measured exposure level compared to measurements performed with sampling duration above 8 h. In the unadjusted model (Table 1), the active sampling method gave a higher measured exposure level compared to passive sampling. The active measurements (10% of all measurements) had a shorter sampling duration compared to passive sampling (median of 110 versus 690 min) and if adjusting for sampling duration, there was no longer any difference in measured exposure levels between the sampling methods.

### Variance components estimates for repeated measurements among workers

In the random effects model, the within-worker variability (_ww_S^2^) was considerably higher compared to the between-installations variability (_bp_S^2^) and the between-worker variability (_bw_S^2^) (Table 4). The design of the process area had the most impact on the between-installation variability and resulted in a 35% reduction in variability. Wind speed explained 14% variability. Job group and season explained 31 and 11% of the between-worker variability in benzene concentration. No factors had mentionable impact on the day-to-day (within-worker) variability. The full model including season, wind speed, design of process area, and job group as fixed factors explained 56% of the between-installation, 39% of the between-worker, and only 1% of the within-worker variability. The reduction in total variability was 18% when using the full model.

**Table 4.** Variance components and explained variance in multilevel tobit mixed-effects models of the log-transformed (ln) personal exposure to benzene (*n* = 294).

Determinants	Between- installation variance	Between-worker within-installation variance	Within-worker variance	Total variance
	_bp_S^2^ (%^*a*^)	_bw_S^2^ (%)	_ww_S^2^ (%)	_bp_S^2^ + _bw_S^2^ + _ww_S^2^ (%)
Random effects model^*b*^	1.25	1.35	4.46	7.06
Mixed-effects models:				
1. Season^*c*^	1.33 (−6.5)	1.20 (10.9)	4.48 (−0.4)	7.01 (0.7)
2. Wind speed^*d*^	1.07 (14.3)	1.96 (−9.38)	4.23 (5.1)	6.77 (4.1)
3. Design of process area^*e*^	0.81 (35.1)	1.30 (3.7)	4.49 (−0.6)	6.60 (6.5)
4. Job group^*f*^	1.20 (4.0)	0.93 (31.2)	4.56 (−2.13)	6.69 (5.2)
5. Season + wind speed + design of process area + job group	0.55 (55.7)	0.83 (38.7)	4.40 (1.2)	5.80 (18.0)

^*a*^% reduction in variance from the random effects model to the respective mixed-effects models.

Total variance _(random effects model)_ – Total variance _(mixed-effects model)_ * 100/Total variance _(random effects model)._

The analogous calculations were conducted for between-worker within-installation (_bw_S^2^), within-worker (_ww_S^2^), and total variance (_bp_S^2^ + _bw_S^2^ + _ww_S^2^).

^*b*^Random effects includes offshore installations and workers.

^*c*^Season: winter and summer.

^*d*^Wind speed: light air (0–3.9 m s^−1^), breeze (4.0–11.9 m s^−1^), and gale (12.0–20.0 m s^−1^).

^*e*^Process area design: open, partially restricted, and restricted process area.

^*f*^Job group^:^ laboratory technicians, mechanics, process operators, industrial cleaners, and others.

## Discussion

The overall measured benzene exposure on 25 Norwegian offshore installation collected from 2002 to 2018 was 0.004 ppm (GM). A time-trend analysis indicated increase in measured benzene exposure over this period. Industrial cleaners had a significantly higher level of exposure than laboratory technicians, mechanics, and process operators. Benzene exposure was higher on partially restricted and restricted installations compared to open installations. Furthermore, exposure in summertime was higher than in wintertime, with a decreased exposure with increasing wind speed (Table 1).

The annual increase of 7.6% in measured benzene exposure was surprising and is not in line with most other studies of occupational exposure showing an overall decrease in exposure. Different industries show annual reductions in exposure levels of around 2–10% (Steinsvåg *et al.*, 2005; Creely *et al.*, 2007; De Vocht *et al.*, 2007; Galea *et al.*, 2009; Kauppinen *et al.*, 2013; Peters *et al.*, 2016; Baldwin *et al.*, 2019; Hidajat *et al.*, 2019). A downward trend was also reported for exposure to oil vapour and mist in offshore drilling areas with an annual reduction of 6% and 8%, respectively (Steinsvåg *et al.*, 2005). However, for the period 1994–2003, Steinsvåg *et al.* (2007) reported GM of benzene exposure at 0.007 ppm for offshore workers on the Norwegian Continental shelf (NCS). This is higher than our findings of 0.004 ppm, indicating that benzene exposure was higher before rather than after 2003.

In our study, a possible change in the measurement strategy might have contributed to the increase in measured benzene exposure level over time. In the present study, the period 2002–2006 included measurements mainly performed on random days in normal operation condition (Kirkeleit *et al.*, 2006; Bråtveit *et al.*, 2007; Steinsvåg *et al.*, 2007). The period from 2007 to 2011 contained measurements that were mainly from the project ‘Chemical working environment in the oil and gas industry’ initiated by Norwegian Oil and Gas Association where the objectives were to share new knowledge within the industry on measured exposure levels and focussing on work operations with potentially high benzene exposure. In addition, control measures to reduce chemical exposure, including the use of appropriate personal protective equipment (PPE) among offshore workers were shared (Norwegian Oil and Gas Association, 2011). Consequently, the measurement strategy was presumably targeted towards workdays that included tasks associated with likely higher exposure. According to the occupational hygiene reports, the same strategy used from 2007 to 2011 was also used from 2012 to 2018. This measurement strategy for benzene is also reported in other studies in the oil industry which may suggest a degree of overestimation of representative full-shift exposure (Panko *et al.*, 2009; Gaffney *et al.*, 2010; Kreider *et al.*, 2010; Swaen *et al.*, 2010). In our study, the decrease in sampling duration over the years (median sampling duration from 2002 to 2006 was 720 min versus 638 min in from 2012 to 2018) supports change in measurements strategy.

The industrial cleaners had the highest exposure to benzene of the investigated job groups. Annual changes for this job group were not possible to estimate since measurements for this job group were only available in 2004 and 2007. The industrial cleaners are mainly employed by contractors. Therefore, measurements performed after 2007 among industrial cleaners have been performed by the contractors themselves and were not available for the present study. Industrial cleaners are included in analyses for overall time trend and if we excluded this group from the analyses, the overall time trend will be slightly lower (7% compared to 7.6%). About one half of the measurements among the industrial cleaners were performed during cleaning of tanks, which has previously been described as the work task with potential for the highest exposure (Kirkeleit *et al.*, 2006; Panko *et al.*, 2009; Gaffney *et al.*, 2010). However, according to the measurements included in Kirkeleit *et al.* (2006), the industrial cleaners used PPE such as facemask with combination filter, chemical suits, gloves, and rubber boots. We do not have any information of what type of PPE or to what extent it was used in measurements in 2007.

The overall annual decrease in measured exposure among laboratory technician was 12.7%. From 2012 to 2018, 6 out of 12 measurements were performed during sampling of ballast water with a low benzene content. If we exclude the six measurements when sampling ballast water, the overall decrease is less than 1%, and no longer statistically significant. We might also assume that measures established after the ‘chemical project’ such as the construction of closed sampling systems and new and better extraction cabinets for the chemical analysis of samples, have reduced the level of exposure. Moreover, it is expected that the awareness of benzene exposure has increased over time and working habits may therefore have changed, for instance by choosing more favourable positions regarding wind direction when flushing and taking samples from the process stream. However, due to few measurements on laboratory technicians from 2012 to 2018, it is not possible to investigate any change in the level of exposure resulting from such measures.

An annual increase in exposure to benzene of 16.8% was observed for the mechanics when including only measurements with a sampling duration above 8 h. Mechanics’ exposure to benzene prior to working on the process system depends on how the equipment or pipes are prepared for maintenance. In most cases, flushing with water before opening, ventilation after opening, and cleaning of equipment after disassembling will reduce the level of exposure for mechanics. Awareness of tasks with potential high exposure that typically falls under the mechanics work area has increased over the years, being reflected in the number of measurements collected for this job group. In the period 2002–2006, only 23% of all measurements were conducted on mechanics. In 2007–2011, 31% and in 2012–2018, 45% of all measurements were performed on mechanics. Hence, more attention to mechanics’ level of exposure might have increased knowledge about tasks with a potentially high benzene exposure. Thus, the measurements were directed towards days on which these types of tasks took place and therefore might have resulted overestimation of the exposure for this group. Mechanics are also the main contributors to the overall annual increase in exposure. For process operators, the annual increase in exposure was less (6.0%) and not statistically significant but may still indicate a more targeted measurement strategy over time (Table 2). In addition to mechanics and process operators, the job group ‘others’ contribute to the increasing measured benzene exposure over the years.

Benzene exposure was higher during the summer compared to the winter. This could be because wind speed is normally lower in the summer, leading to a decrease in natural ventilation. Wind speed presumably increases the air exchange rate in open process areas. In line with this, the exposure model showed that benzene exposure decreased with increasing wind speeds. However, when adjusting for wind speed, benzene exposure was still lower on open installations compared to restricted and partially restricted installations.

The variance in the random effects model was highest in within-worker compared to between-worker and between-installations. The workers perform a wide range of tasks during their 14-day offshore period and their exposure will consequently vary considerably from one day to the next. However, sufficient information on performed work tasks was not available to estimate the effect on the day-to-day variability in exposure concentrations. The design of the process area had the greatest effect on the between-installation variability and indicates that the extent to which the process area is closed with walls should be considered when assigning exposure levels to job groups in exposure assessments or in epidemiological studies. The job group had the most impact on the between-worker variability. This finding is mainly attributable to the contrast in exposure between industrial cleaners and the other job groups. Both mechanics and process operators had a higher number of measurements during the summer compared to winter which can explain part of the variability between workers.

### Limitations

The data in our study were collected from various exposure measurements assessments. According to the measurements strategies described in the reports, around 23%, all from 2002 to 2006, were done on random days for laboratory technicians, mechanics, and process operators. However, in the subsequent years, the measurements were conducted on days assigned to tasks that were known to be associated with benzene exposure, which may reflect the large number of measurements with a short sampling duration (Fig. 1). This strategy might be preferable when the aim of the assessment is to provide information on whether preventive measures are required to reduce the level of exposure. Moreover, this sampling strategy may have led to an overestimation of the full-shift, daily exposure for these job groups. However, the average benzene exposures were close to the LOD which might have decreased the precision of the estimated exposure levels.

A worker can be exposed to benzene from various sources during their work shift. The content of the production stream might affect exposure since the benzene content vary between sources, such as crude oil [0.01–0.90 percentage by weight (wt%) benzene], condensates (0.42 and 1.98 wt%) (Gjesteland *et al.*, 2019), produced water (<0.1 wt%), and wet glycol (0.1–1 wt%) (Norwegian Oil and Gas Association, 2014). Due to a lack of detailed information about sources of exposure, the benzene source was not included as a determinant in the study.

The present study is limited to measurements of ambient benzene exposure, and although the use of both respiratory and skin protection has increased during the study period, we do not have any specific information on the use of PPE. Due to the high volatility of benzene, occupational exposure to benzene mainly occurs via inhalation (IARC, 2018). Nevertheless, dermal contact with benzene source might contribute to the total exposure burden for some tasks (i.e. tank cleaning operations or equipment containing residues of crude oil, using petroleum-based products as degreasing agents). However, the dermal absorption of benzene will depend on the benzene content and composition of the source, contact time, and the area of the body on which the chemical resides (IARC, 2018).

From 2012 to 2015, only three measurements with a sampling duration above 60 min were performed and two of these measurements were below the LOD. Hence, knowledge about benzene exposure during this period is insufficient.

## Conclusions

There was an overall annual increase in measured benzene exposure during 2002–2018. One reason might be an increased fraction of measurements taken on workdays with tasks known to be associated with benzene exposure. Increased exposure for mechanics was specially observed for measurements lasting 8 h or more. No statistically significant changes in measured exposure were observed for process operators. A decrease in measured exposure level was observed for laboratory technicians, but due to a limited number of measurements after 2011, no conclusions could be drawn. Industrial cleaners had the highest measured benzene exposure among the investigated job groups. However, since the industrial cleaners use full PPE when performing highly exposed tasks such as tank cleaning, their actual exposure leading to benzene uptake might be lower than the estimated exposure. Open process areas, high wind speed (>12 m s^−1^), and winter appeared to be strongly associated with lower exposure to benzene. Job group, design of process area, season, and wind speed need to be considered as important determinants of exposure to benzene in Norwegian offshore petroleum industry.

## Data Availability

The data underlying this article are not shared publicly to protect the privacy of organizations that participated in the study.

## References

[CIT0001] Almerud P , Akerstrom M, Andersson EM et al (2017) Low personal exposure to benzene and 1,3-butadiene in the Swedish petroleum refinery industry. Int Arch Occup Environ Health; 90: 713–24. 28578463 10.1007/s00420-017-1234-y PMC5583277

[CIT0002] Baldwin PEJ , Yates T, Beattie H et al (2019) Exposure to respirable crystalline silica in the GB brick manufacturing and stone working industries. Ann Work Expo Health; 63: 184–96. 30624605 10.1093/annweh/wxy103

[CIT0003] Bråtveit M , Hollund B, Kirkeleit J et al (2012) Supplementary information to the Job Exposure Matrix for benzene, asbestos and oil mist/oil vapour among Norwegian offshore workers. Bergen, Norway: University of Bergen and Uni Health.

[CIT0004] Bråtveit M , Kirkeleit J, Hollund BE et al (2007) Biological monitoring of benzene exposure for process operators during ordinary activity in the upstream petroleum industry. Ann Occup Hyg; 51: 487–94. 17607018 10.1093/annhyg/mem029

[CIT0005] Committee for Risk Assessment (RAC). (2018) Opinion on scientific evaluation of occupational exposure limits for Benzene. https://echa.europa.eu/documents/10162/13641/benzene_opinion_en.pdf/4fec9aac-9ed5-2aae-7b70-5226705358c7. Accessed 5 May 2020.

[CIT0006] Creely KS , Cowie H, Van Tongeren M et al (2007) Trends in inhalation exposure—a review of the data in the published scientific literature. Ann Occup Hyg; 51: 665–78. 17932083 10.1093/annhyg/mem050

[CIT0007] De Vocht F , Burstyn I, Straif K et al (2007) Occupational exposure to NDMA and NMor in the European rubber industry. J Environ Monitor; 9: 253–9. 10.1039/b615472g 17344951

[CIT0008] Det Norske Veritas Germanischer Lloyd (DNVGL). (2016) Standard: offshore substations. 6.4.2 Design process. Norway: Det Norske Veritas Germanischer Lloyd. Available at: http://rules.dnvgl.com/docs/pdf/dnvgl/ST/2016-04/DNVGL-ST-0145.pdf. Accessed 25 February 2021.

[CIT0009] European Chemicals Agency (ECHA). (2018) Background document in support of the Committee for Risk Assessment (RAC) evaluation of limit values for benzene in the workplace. Helsinki, Finland: European Chemicals Agency.

[CIT0010] Gaffney SH , Burns AM, Kreider ML et al (2010) Occupational exposure to benzene at the ExxonMobil refinery in Beaumont, TX (1976–2007). Int J Hyg Environ Health; 213: 285–301. 20494616 10.1016/j.ijheh.2010.04.004

[CIT0011] Galea KS , Van Tongeren M, Sleeuwenhoek AJ et al (2009) Trends in wood dust inhalation exposure in the UK, 1985–2005. Ann Occup Hyg; 53: 657–67. 19602501 10.1093/annhyg/mep044

[CIT0012] Gjesteland I , Hollund BE, Kirkeleit J et al (2019) Determinants of airborne benzene evaporating from fresh crude oils released into seawater. Mar Pollut Bull; 140: 395–402. 30803659 10.1016/j.marpolbul.2018.12.045

[CIT0013] Glass DC , Gray CN, Jolley DJ, et al (2003) Leukemia risk associated with low-level benzene exposure. Epidemiology; 14: 569–77. 14501272 10.1097/01.ede.0000082001.05563.e0

[CIT0014] Helsel DR . (2005) Nondetects and data analysis: statistics for censored environmental data. Hoboken, NJ: Wiley.

[CIT0015] Hidajat M , Mcelvenny DM, Mueller W, et al (2019) Job-exposure matrix for historical exposures to rubber dust, rubber fumes and *N*-nitrosamines in the British rubber industry. Occup Environ Med; 76: 259–67. 30772817 10.1136/oemed-2018-105182 PMC6581116

[CIT0016] International Agency for Research on Cancer (IARC). (2018) Benzene. In: IARC, editor. IARC monographs on the evaluation of the carcinogenic risks to humans. Vol. 120. Lyon, France: IARC.

[CIT0017] Kauppinen T , Uuksulainen S, Saalo A et al (2013) Trends of occupational exposure to chemical agents in Finland in 1950–2020. Ann Occup Hyg; 57: 593–609. 23230130 10.1093/annhyg/mes090

[CIT0018] Kirkeleit J , Riise T, Bråtveit M et al (2006) Benzene exposure on a crude oil production vessel. Ann Work Expo Health; 50: 123–9. 10.1093/annhyg/mei065 16371415

[CIT0019] Kreider ML , Unice KM, Panko JM et al (2010) Benzene exposure in refinery workers: ExxonMobil Joliet, Illinois, USA (1977–2006). Toxicol Ind Health; 26: 671–90. 20643709 10.1177/0748233710378115

[CIT0020] Lotz A , Kendzia B, Gawrych K et al (2013) Statistical methods for the analysis of left-censored variables [Statistische Analysemethoden für linkszensierte Variablen und Beobachtungen mit Werten unterhalb einer Bestimmungs- oder Nachweisgrenze]. GMS Medizinische Informatik, Biometrie und Epidemiologie; 9: Doc05.

[CIT0021] Norwegian Oil and Gas Association. (2011) Kjemisk arbeidsmiljø i den norske petroleumsindustrien. Stavanger, Norway: Norwegian Oil and Gas Association.

[CIT0022] Norwegian Oil and Gas Association. (2014) 131—Norsk olje og gass Anbefalte retningslinjer for identifisering, vurdering, kontroll og oppfølging av benzeneksponering. Norway: Norwegian Oil and Gas Association.

[CIT0023] Panko JM , Gaffney SH, Burns AM et al (2009) Occupational exposure to benzene at the ExxonMobil refinery at Baton Rouge, Louisiana (1977–2005). J Occup Environ Hyg; 6: 517–29. 19544135 10.1080/15459620903044161

[CIT0024] Peters S , Vermeulen R, Portengen L et al (2016) SYN-JEM: a quantitative job-exposure matrix for five lung carcinogens. Ann Occup Hyg; 60: 795–811. 27286764 10.1093/annhyg/mew034

[CIT0025] Ridderseth H , Ousman N, Hollund BE et al (2019) Benzene exposure among selected job categories on offshore oil installations. Environ Epidemiol; 3: 40.

[CIT0026] Steinsvåg K , Bråtveit M, Moen BE. (2005) Exposure to oil mist and oil vapour during offshore drilling in Norway, 1979–2004. Ann Occup Hyg; 50: 109–22. 16141252 10.1093/annhyg/mei049

[CIT0027] Steinsvåg K , Bråtveit M, Moen BE. (2007) Exposure to carcinogens for defined job categories in Norway’s offshore petroleum industry, 1970 to 2005. Occup Environ Med; 64: 250–8. 17043075 10.1136/oem.2006.028225 PMC2078458

[CIT0028] Stenehjem JS , Kjærheim K, Bråtveit M et al (2015) Benzene exposure and risk of lymphohaematopoietic cancers in 25 000 offshore oil industry workers. Br J Cancer; 112: 1603–12. 25867262 10.1038/bjc.2015.108 PMC4453669

[CIT0029] Swaen GMH , Van Amelsvoortltwisk JJ, Verstraeten E et al (2010) Low level occupational benzene exposure and hematological parameters. Chem Biol Interact; 184: 94–100. 20074561 10.1016/j.cbi.2010.01.007

[CIT0030] Verma DK , Johnson DM, Mclean JD. (2000) Benzene and total hydrocarbon exposures in the upstream petroleum oil and gas industry. Am Ind Hyg Assoc; 61: 255–63. 10.1080/15298660008984534 10782197

